# GSK‐3β activation index is a potential indicator for recurrent inflammation of chronic rhinosinusitis without nasal polyps

**DOI:** 10.1111/jcmm.13274

**Published:** 2017-07-17

**Authors:** Haiyu Hong, Fenghong Chen, Yongkang Qiao, Yan Yan, Rongkai Zhang, Zhe Zhu, Huabin Li, Yunping Fan, Geng Xu

**Affiliations:** ^1^ Department of Otolaryngology and Head Neck Surgery of the First Hospital Affiliated with Sun Yat‐sen University Guangzhou Guangdong China; ^2^ Department of Otolaryngology and Head Neck Surgery of the Fifth Hospital Affiliated with Sun Yat‐sen University Zhuhai Guangdong China; ^3^ Department of Physiology Yong Loo Lin School of Medicine National University of Singapore Singapore City Singapore; ^4^ Department of Otolaryngology Yong Loo Lin School of Medicine National University of Singapore Singapore City Singapore; ^5^ Department of Stem Cell Biology and Regenerative Medicine Lerner Research Institute Cleveland Clinic Cleveland OH USA; ^6^ Department of Otolaryngology Head and Neck Surgery Affiliated Eye, Ear, Nose and Throat Hospital Fudan University Shanghai China

**Keywords:** chronic rhinosinusitis without nasal polyps, GSK‐3β, nuclear factor‐κB, phosphorylation

## Abstract

Chronic rhinosinusitis without nasal polyps (CRSsNP) is one of the most common otorhinolaryngologic diseases worldwide. However, the underlying mechanism remains unclear. In this study, the expression of glycogen synthase kinase 3 (GSK‐3) was quantitatively evaluated in patients with CRSsNP (*n* = 20) and healthy controls (*n* = 20). The mRNA levels of GSK‐3α and GSK‐3β were examined by qPCR, the immunoreactivities of GSK‐3β and nuclear factor‐κB (NF‐κB) were examined by immunohistochemistry (IHC) staining, and the protein levels of GSK‐3β, phospho‐GSK‐3β (p‐GSK‐3β, s9) and NF‐κB were examined using Western blot analysis. We found that GSK‐3 was highly expressed in both CRSsNP and control groups without significant difference in both GSK‐3β mRNA and protein levels. However, when compared with healthy control group, the GSK‐3β activation index, defined as the ratio of GSK‐3β over p‐GSK‐3β, was significantly decreased, whereas the NF‐κB protein abundance was significantly increased in CRSsNP group (*P* < 0.05). Strikingly, the GSK‐3β activation index, was highly correlated with NF‐κB protein level, as well as CT scores in CRSsNP group (*P* < 0.05). It was also highly correlated with the mRNA expressions of inflammation‐related genes, including T‐bet, IFN‐γ and IL‐4 in CRSsNP group (*P* < 0.05). Our findings suggest that GSK‐3β activation index, reflecting the inhibitory levels of GSK‐3β through phosphorylation, may be a potential indicator for recurrent inflammation of CRSsNP, and that the insufficient inhibitory phosphorylation of GSK‐3β may play a pivotal role in the pathogenesis of CRSsNP.

## Introduction

CRSsNP is one of the most common otorhinolaryngologic diseases worldwide, with an estimated prevalence of 8% in China 1, 10.9% in Europe 2 and 12.5% in USA 3. CRS is commonly subdivided into two types: CRS with nasal polyps (CRSwNP) and CRS without nasal polyps (CRSsNP) 4. Epidemiologic evidence has suggested an association between CRSsNP prevalence and air pollution, smoking, perennial allergic rhinitis, asthma 3, 5. The underlying pathogenic mechanism of CRSsNP remains unclear.

GSK‐3 is a highly conserved serine/threonine protein kinase found in all eukaryotes, which exists in two isoforms, namely GSK‐3α and GSK‐3β 6. GSK‐3β is implicated in several diseases, such as neurodegenerative diseases, cancer, inflammation and cardiovascular disease 7, 8, 9. Recently, some reports found that GSK‐3β might be involved in the pathogenesis of CRSwNP, and the underlying mechanisms remain unknown 9, 10.

The transcription factor NF‐κB is activated by a variety of cellular and developmental signals 11. Excessive activation of NF‐κB signalling pathway has been proposed as the central event in the pathogenesis of inflammatory response 12, 13, 14. The regulatory role of GSK‐3β on NF‐κB and inflammatory response have recently been established in the immune response, cell survival and cancer. Inhibition of GSK‐3β activity clearly leads to inhibition of NF‐κB activation, and this in turn results in potent anti‐inflammatory effects in several animal models of inflammatory disorders 15, 16, 17. However, whether GSK‐3β activity was involved in the pathogenesis of CRSsNP has yet to be completely understood.

## Materials and methods

### Subjects

This study was approved by the local ethics committee boards, and all subjects provided written informed consent. We enrolled 20 patients with CRSsNP and 20 control subjects in this study from the Department of Otolaryngology, the First Affiliated Hospital of Sun Yat‐sen University. The diagnoses of CRSsNP in this study were based on the patient's clinical characteristics and computed tomography (CT) scan of the paranasal cavities, in accordance with the European position paper on rhinosinusitis and nasal polyps 2012 (EPOS 2012) guidelines 4. The controls were patients undergoing transnasal optic nerve decompression because of traumatic neuropathy who were free of nasal symptoms. The demographic data of all subjects enrolled in the study were listed in Table 1.

**Table 1:** Patients' characteristics

	Normal	CRS	*P* value
Total no. of subjects	20	20	
Gender (M/F)	15/5	12/8	0.3112
Age (years), mean (S.D.)	33.25 (13.81)	34.7 (12.76)	0.7302
Atopy, no. (%)	2 (10.00)	4 (20.00)	0.6579
Asthma, no.	0	1	1
Smoking, no. (%)	4 (20.00)	3 (15.00)	0.6773
CT Scores, mean(S.D.)	N/A	5.8 (2.95)	

CRSsNP, chronic rhinosinusitis without nasal polyps; CT, computed tomography; N/A, not applicable.

Statistical analysis of age was performed by the Mann–Whitney *U*‐test, and those of gender, atopy, asthma and smoking were performed by the chi‐square test.

Uncinate process tissues (UTs) were harvested during surgery and were further used for quantitative reverse transcription polymerase chain reaction (qPCR), IHC staining and Western blot analysis.

### qPCR

Total RNA was extracted from UTs with TRIzol reagent (Invitrogen, Carlsbad, CA, USA) according to the manufacturer's instructions. Reverse transcription (RT) was performed, according to the manufacturer's protocol by ReverTra Ace qPCR RT Kit (Toyobo Biochemicals, Osaka, Japan). qPCR was performed as we previously reported 7. The sequences of the primer were as follows: GAPDH forward: 5′‐ GAA ATC CCA TCA CCA TCT TCC A ‐3′, GAPDH reverse: 5′‐ TGG ACT CCA CGA CGT ACT C ‐3′; GSK‐3α forward: 5′‐ TTT GAT GAA CTG CGA TGT CTG ‐3′, GSK‐3α reverse: 5′‐ GGA TCA GAA TGG CGT TGA G ‐3′; GSK‐3β forward: 5′‐ CTG TTC CGA AGT TTA GCC TAT AT ‐3′, GSK‐3β reverse: 5′‐ ACA AGA GGT TCT GCG GTT TA ‐3′; T‐bet forward: 5′‐ CGG CTG CAT ATC GTT GAG GT ‐3′, T‐bet reverse: 5′‐ GCA GTC ACG GCA ATG AAC TG ‐3′; GATA‐3 forward: 5′‐ TCA TTA AGC CCA AGC GAA GG ‐3′, GATA‐3 reverse: 5′‐ GTC CCC ATT GGC ATT CCT C ‐3′; RORc forward: 5′‐ CAT GTC CCG AGA TGC TGT CA ‐3′, RORc reverse: 5′‐ GAC CAC TGG TTC CTG TTG CTG ‐3′; IL‐4 forward: 5′‐ TTG CTG CCT CCA AGA ACA CAA CTG ‐3′, IL‐4 reverse: 5′‐ TTC CTG TCG AGC CGT TTC AGG AAT ‐3′; IL‐5 forward: 5′‐ TGC TGA TAG CCA ATG AGA CTC TG ‐3′, IL‐5 reverse: 5′‐ TTT CCA CAG TAC CCC CTT GC ‐3′; IL‐13 forward: 5′‐ TGG TCA ACA TCA CCC AGA ACC AGA ‐3′, IL‐13 reverse: 5′‐ AGC CTG ACA CGT TGA TCA GGG ATT ‐3′; IFN‐γ forward: 5′‐ TGC AGG TCA TTC AGA TGT AGC GGA ‐3′, IFN‐γ reverse: 5′‐ TGT CTT CCT TGA TGG TCT CCA CAC TC ‐3′; IL‐17A forward: 5′‐ CAA CCG ATC CAC CTC ACC ‐3′, IL‐17A reverse: 5′‐ AGC CCA CGG ACA CCA GTA ‐3′. All experiments were performed in triplicate for each data point. Melting curve analysis was used to control for amplification specificity. Expression of target gene was expressed as fold increase or decrease relative to the expression of GAPDH. The mean value of the replicates for each sample was calculated and expressed as cycle threshold (Ct). The amount of gene expression was then calculated as the difference (ΔCt) between the Ct value of target gene and the Ct value of GAPDH. Fold changes in target gene mRNA were determined as 2^−ΔΔCt^.

### IHC staining

UTs were collected from 20 CRSsNP patients and 20 controls as mentioned above. IHC staining was performed using the Envision method. A mouse anti‐human GSK‐3β monoclonal antibody (Abcam, Cambridge, MA, USA) and a mouse anti‐human NF‐κB were all used at a dilution of 1:250 according to the manufacturer's instructions. Each of these sections was incubated with a secondary antibody and then with horse radish peroxidase‐labelled streptavidin complex (Zhong‐Shan‐Jin‐Qiao, Beijing, China). Distribution of peroxidase was revealed by incubating the sections in a solution containing 3% 3,3′‐diaminobenzidine tetrahydrochloride before being counterstained with haematoxylin and cover slipped. Negative control studies were performed using normal immunoglobulin G to replace the primary antibody in appropriate concentration. The histological analyses were performed in double‐blind manner. The IHC intensities of five randomly selected epithelial areas (400×) on each section of the subjects were quantitatively scored using Image‐Pro Plus 6.0 software and averaged.

### Western blot analysis

Total protein was extracted in 100 μl of RIPA lysis buffer per 100 mg UTs at 4°C for 30 min. The protein concentration was determined by the Bradford method. Samples containing 10 μg of protein were boiled, subjected to SDS‐PAGE in 10% Tris‐glycine gels and transferred electrophoretically to a polyvinylidene fluoride membrane. The membrane was incubated with 5% fat‐free skim milk in Tris‐buffered solution (TBS) containing 0.05% Tween 20 (1 hr, room temperature) and then incubated with mouse anti‐human primary antibodies (GSK‐3β, 1:5000; p‐GSK‐3β, 1:10,000; NF‐κB, 1:3000, all were purchased from Abcam; GAPDH, 1:10,000, Cell Signaling, Danvers, MA, USA) (overnight, 4°C). The membrane was then incubated with horseradish peroxidase‐linked secondary antibody (goat anti‐mouse immunoglobulin G, 1:1000), processed using the ECL chemiluminescence reaction kit (Cell Signaling), and then followed by exposure on medical film. The relative band densities of target proteins compared with GAPDH were quantified with the Bio‐Rad Quantity One 1‐D Analysis Software.

### Statistical analysis

Data were expressed as the median and interquartile range (IQR), and analysed using non‐parametric Mann–Whitney *U*‐test for continuous variable and chi‐square test for dichotomous variable. A *P* value of less than 0.05 was considered statistically significant.

## Results

### Demographic characteristics of study population

This study enrolled 40 subjects from the Department of Otolaryngology, the First Affiliated Hospital of Sun Yat‐sen University. Of them, 20 were CRSsNP patients, with age ranging from 16 to 55 years (mean age: 34.7 ± 12.56 years, 12 males), and 20 were patients undergoing transnasal optic nerve decompression because of traumatic neuropathy who were free of nasal symptoms that was recruited as control group, with age ranging from 17 to 59 years (mean age: 33.25 ± 13.81 years, 15 males). The demographic characteristics of study population were summarized in Table 1.

### GSK mRNA expression and immunoreactivity in CRSsNP

**Figure 1:**
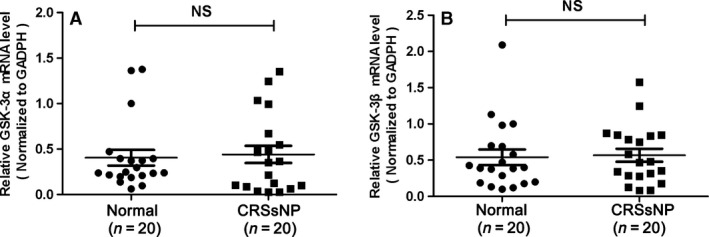
The mRNA expression of GSK‐3α and GSK‐3β in CRSsNP and normal control. (**A**) GSK‐3α mRNA expression in CRSsNP and normal control; (**B**) GSK‐3β mRNA expression in CRSsNP and normal control. The data indicated the median (IQR) of CRSsNP and normal control.

**Figure 2:**
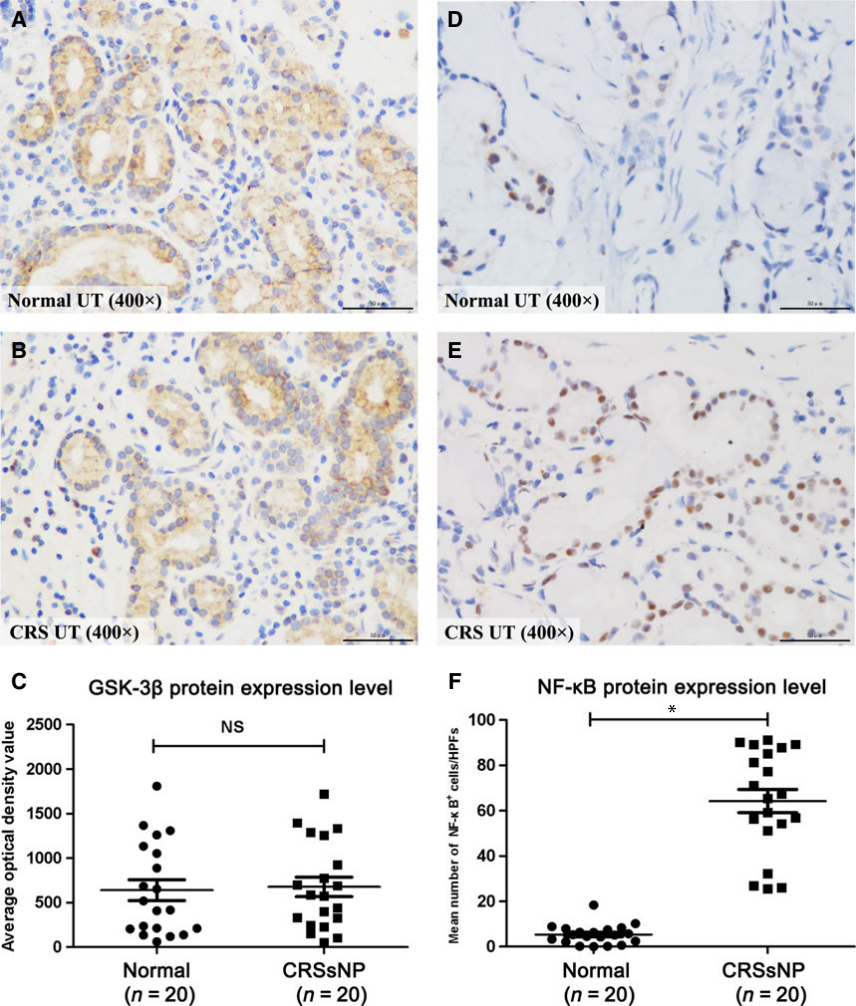
The immunoreactivity of GSK‐3β and NF‐κB in CRSsNP and normal control. (**A** and **B**) Representative IHC staining of GSK‐3β and NF‐κB in CRSsNP and normal control was shown (400×); (**C** and **D**), Intensity of GSK‐3β and NF‐κB in CRSsNP and normal control. The data indicated the median (IQR) of CRSsNP and normal control. * *P*<0.05.

To determine the possible role of GSK in the pathogenesis of CRSsNP, we firstly examined the mRNA and protein expression of GSK‐3α and GSK‐3β in two groups. As shown in Figure 1, we found that GSK‐3α and GSK‐3β mRNA were detectable in all samples, but no significant difference of GSK‐3α and GSK‐3β mRNA was observed between CRS and controls. Next, we quantitatively examined the immunoreactivity of GSK‐3β in two groups. As shown in Figure 2A–C, we observed GSK‐3β immunostaining was extensively distributed in both CRSsNP and controls. Semi‐quantitative analysis of the histological sections showed no significant difference of GSK‐3β staining intensity between the two groups.

### The protein levels of GSK‐3β, p‐GSK‐3β (s9) and NF‐κB in CRSsNP

**Figure 3:**
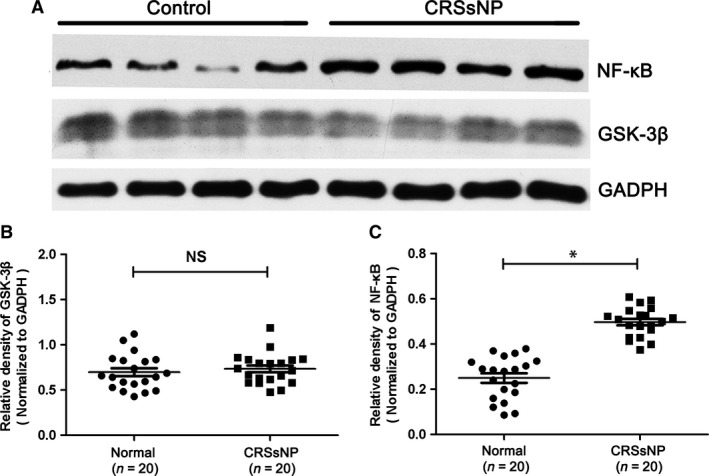
The protein level of GSK‐3β and NF‐κB in CRSsNP and normal control. (**A**) Representative Western blot results of GSK‐3β and NF‐κB in CRSsNP and normal control. (**B**) Relative level of GSK‐3β in CRSsNP and normal control. (**C**) Relative level of NF‐κB in CRSsNP and normal control. * *P*< 0.05

**Figure 4:**
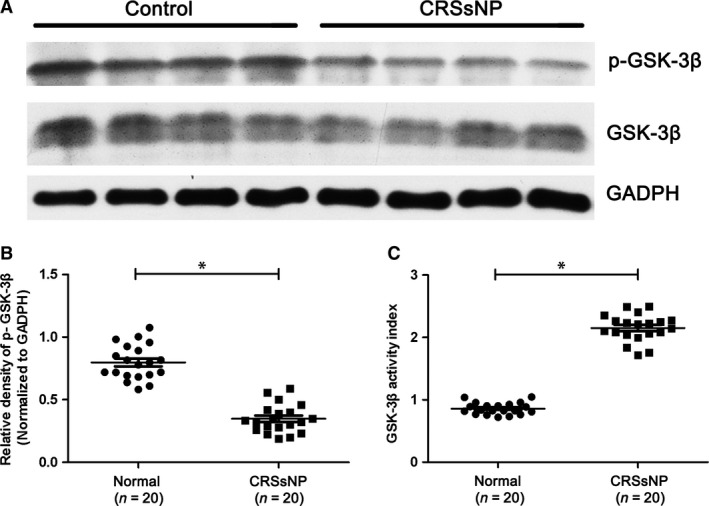
The protein level of phsophorylated GSK‐3β in CRSsNP and normal control. (A) Representative Western blot results of p‐GSK‐3b (s9) and GSK‐3b in CRSsNP and normal control. (B) Relative level of p‐GSK‐3b in CRSsNP and normal control. (C) GSK‐3b activity index in CRSsNP and normal control. * *P*< 0.05.

**Figure 5:**
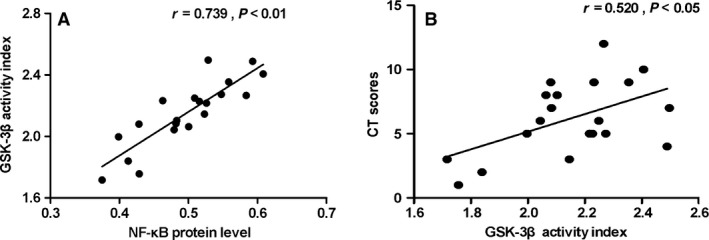
GSK‐3β activity index in CRSsNP. The association of GSK‐3β activity index with the protein level of NF‐κB (**A**) and CT scores (**B**) in CRSsNP.

In the following study, we examined the protein levels of GSK‐3β, p‐GSK‐3β (s9) and NF‐κB in CRSsNP. As shown in Figure 2D–F, we found that the mean numbers of NF‐κB‐positive cells were significantly increased in CRSsNP when compared with controls (*P* < 0.05), with the relative protein level of NF‐κB (normalized to GAPDH level) also significantly increased. In comparison, the total GSK‐3β protein levels remain unchanged in CRSsNP (Fig. 3A–C). However, we found that the phosphorylation level of GSK‐3β (p‐GSK‐3β, s9) (normalized to total GSK‐3β) was significantly decreased in CRSsNP when compared to controls (*P* < 0.05). We defined the GSK‐3β activity index as the ratio of GSK‐3β over p‐GSK‐3β (GSK‐3β/p‐GSK‐3β). The results showed that GSK‐3β activity index was significantly increased in CRSsNP comparing with controls (*P* < 0.05) (Fig. 4), and GSK‐3β activity index and NF‐κB protein abundance were highly correlated in CRSsNP (*r* = 0.739, *P* < 0.01) (Fig. 5A). As shown in Figure 5B, we also found that there was a positive association between GSK‐3β activity index and CT scores.

### GSK‐3β activity index and the mRNA expression of inflammatory cytokines

**Figure 6:**
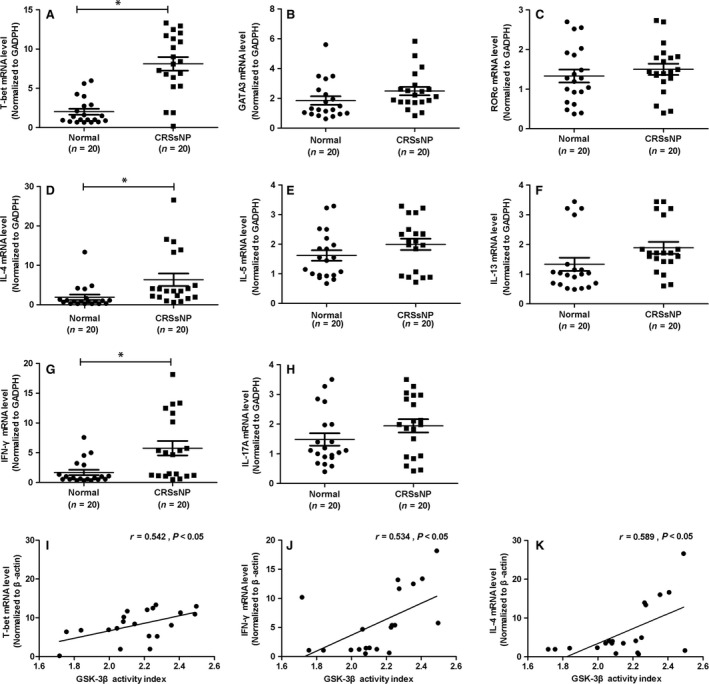
GSK‐3β activity index and chronic inflammation in CRSsNP. mRNA expression of inflammatory‐related cytokines, including T‐bet (**A**), GATA3 (**B**), RORc (**C**), IL‐4 (**D**), IL‐5 (**E**), IL‐13 (**F**), IFN‐γ (**G**) and IL‐17A (**H**) in CRSsNP compared to controls. Correlation analysis between GSK‐3β activity index and the inflammation‐related genes, including T‐bet (**I**), IFN‐γ (**J**) and IL‐4 (**K**). * *P*< 0.05.

To evaluate the relationship between GSK‐3β activity index and chronic inflammation in CRSsNP, we detected the mRNA expression of inflammation‐related genes (T‐bet, RORC, GATA3, IFN‐γ, IL‐4, IL‐5, IL‐13, and IL‐17A). Our results showed mRNA levels of T‐bet, IFN‐γ and IL‐4 were significantly increased in CRSsNP than those in controls. In the following correlation analysis, we found that there was a positive association between GSK‐3β activity index and the inflammation‐related genes, including T‐bet, IFN‐γ, and IL‐4 (Fig. 6).

## Discussion

In this study, we provided the first evidence that GSK‐3α and 3β were highly expressed in both normal and CRSsNP nasal mucosa. Moreover, we found that the GSK‐3β activation index (GSK‐3β/p‐GSK‐3β) was significantly correlated with NF‐κB protein level in CRSsNP group, which may be a potential indicator for recurrent inflammation of CRSsNP. These findings, therefore, expand our understanding on the pathogenesis of CRSsNP and demonstrate their potential use as therapeutic interventions.

GSK‐3β is a crucial regulator of the balance between pro‐inflammatory and anti‐inflammatory cytokine production, which has been investigated for its signalling pathway and protein function 18. GSK‐3β suppresses the transcriptional activity of cAMP response element‐binding protein (CREB) and activator protein 1 (AP‐1), both of which contribute to IL‐10 expression, and that can dampen the production of anti‐inflammatory IL‐10 19. These discoveries led to the rapid application of GSK‐3β inhibitors in animal disease models of colitis, arthritis, multiple sclerosis and others 20. In a recent study on CRSwNP, the positive correlation was found among the expression of GSK‐3β, PI3K/Akt and IL‐6 in CRSwNP, which suggested that GSK‐3β may play a pro‐inflammatory role in the occurrence and development of CRSwNP 21. Previously, we reported that GSK‐3β inhibition was related with the suppressive activity of Treg cells in nasal polyps, but the underlying molecular mechanism is unknown 10. In this study, we have firstly demonstrated that either GSK‐3α or GSK‐3β is highly expressed in both the CRSsNP and normal nasal mucosa, indicating that GSK‐3 may be involved in the inflammatory response in CRSsNP patients. However, we failed to observe any significant difference in GSK‐3α and GSK‐3β mRNA levels between CRSsNP and normal groups.

Unlike most protein kinases, GSK‐3β is constitutively active in resting cells and involved in diverse intracellular signalling in response to the external signals. GSK‐3β activity is regulated by site‐specific phosphorylation. It is known that phosphorylation at serine (s9) inhibits GSK‐3β activity 6. GSK‐3β is subjected to multiple regulatory mechanisms, and phosphorylation of s9 is probably the most important regulatory mechanism. Our results revealed a significantly higher NF‐κB expression in CRSsNP group compared with normal group, indicating enhanced inflammatory response in the nasal mucosa of CRSsNP group. NF‐κB is activated by a variety of cellular and developmental signals, and GSK‐3β has been proposed as the regulator of NF‐κB signalling pathway in various inflammatory responses. Although the exact molecular mechanism of how GSK‐3β activates NF‐kB remains unclear, there is increasing evidence from ours and others' studies that inhibition of the activity of GSK‐3β may affect multiple steps in the cascade of events leading to the activation of NF‐κB 10, 22, 23. In present study, we found that decreased p‐GSK‐3β expression in CRSsNP group compared with normal group, suggesting insufficient inhibition of GSK‐3β by phosphorylation may contribute to the enhanced NF‐κB signalling pathway in CRSsNP patients.

Consistently, we found GSK‐3β activation index (ratio of GSK‐3β over p‐GSK‐3β) was positively associated with NF‐κB protein level in CRSsNP patients. Interestingly, we also found that there was a positive association between GSK‐3β activity index and CT scores. Next, we detected the mRNA expression of inflammation‐related genes, and also found the mRNA levels of transcription factor T‐bet, as well as inflammation cytokines IFN‐γ and IL‐4 were significantly increased in CRSsNP compared to controls. These results indicate that chronic inflammation may be one of key determinants in the pathogenesis of CRSsNP. Additionally, in the following correlation analysis, GSK‐3β activity index showed a positive association with all these inflammation‐related genes, including T‐bet, IFN‐γ, and IL‐4. Our findings suggested that GSK‐3β activation index, reflecting inhibitory level of GSK‐3β through phosphorylation, may be a potential indicator for recurrent inflammation of CRSsNP.

We observed inactivation of GSK‐3β and activation of NF‐kB in nasal mucosa of CRSsNP patients. Taken together, these findings can expand our understanding on the pathophysiology of CRSsNP and contributed to development of novel therapeutic target. Further systematic studies are needed to elucidate the significance of the changes found in this single enzyme on the overall system of cell signalling in CRSsNP.

## Conclusion

We demonstrated that although GSK‐3 is highly expressed in both normal and CRSsNP groups, the phosphorylation level of p‐GSK‐3β was significantly decreased in CRSsNP group. The GSK‐3β activation index (ratio of GSK‐3β over p‐GSK‐3β) was positively correlated with NF‐κB protein level in CRSsNP group (*P* < 0.05). Our findings suggest that GSK‐3β activation index is a potential indicator for recurrent inflammation of CRSsNP, and the insufficient inhibition of GSK‐3β by phosphorylation may play a pivotal role in the pathogenesis of CRSsNP.

## Conflict interests

The authors declare that they have no competing interest.
